# Sociocultural factors influencing alcohol use among Korean immigrant women: A scoping review

**DOI:** 10.1371/journal.pone.0353742

**Published:** 2026-07-14

**Authors:** Catherine Kim, Michael Butac, Hyoun S. Kim, Rosanra Yoon, Josephine Pui-Hing Wong

**Affiliations:** 1 Faculty of Community Services, Toronto Metropolitan University, Toronto, Ontario, Canada; 2 Department of Psychology, Toronto Metropolitan University, Toronto, Ontario, Canada; 3 Faculty of Nursing, University of Toronto, Toronto, Ontario, Canada; University of Huelva: Universidad de Huelva, SPAIN

## Abstract

**Background:**

Although studies of alcohol use have explored the experiences of various ethnocultural communities in diverse countries such as Canada, there is a noticeable gap in primary research concerning the experiences of Korean immigrant women. With alcohol consumption rising among women relative to men in recent decades, a critical understanding of existing knowledge on alcohol use through the lenses of culture and gender is essential.

**Research Question:**

What is the current state of evidence on alcohol use among Korean immigrant women?

**Methods:**

This scoping review was conducted using the five-stage framework adapted from Arksey and O’Malley (2005) and extended by Levac et al. (2010), with reporting guided by the Joanna Briggs Institute PRISMA-ScR framework. Inclusion criteria comprised primary studies focused on both Korean immigrant women and alcohol use, from Canada, the United States, the United Kingdom, Australia, and New Zealand, restricted to adult ages and English-language publications. Inductive thematic analysis as outlined by Gottlieb et al. (2021) was employed to extract themes from the included articles.

**Results:**

Seven articles met the inclusion criteria, all from the United States, revealing no existing research on this topic from any other eligible country. Three major sociocultural themes influencing alcohol use were identified: acculturation, religion, and social networks. Additional contextual factors including immigration-related stress and traditional Korean drinking customs were noted as plausible but less consistently reported influences.

**Conclusions:**

Findings underscore the importance of adopting culturally sensitive, gender-specific approaches in public health initiatives targeting alcohol use among Korean immigrant women. Substantial gaps remain, particularly regarding the Canadian context.

## Introduction

Over recent decades, ethnic diversity has expanded considerably in countries such as Canada, with immigration playing a pivotal role in shaping social and cultural landscapes. Within these growing multicultural communities, Koreans have emerged as a dynamic and expanding population, with immigration numbers increasing notably since the late 20th century [1]. Korean immigrants predominantly settle in major urban centres across destination countries, with Toronto and Vancouver leading in Canada [2], Sydney in Australia [3], Los Angeles, New York, and Washington D.C. in the United States [4], London in England [5,6], and Auckland in New Zealand [7]. This pattern reflects a broader tendency for Korean migrants to concentrate in key metropolitan hubs where cultural, business, and social networks are established.

Despite this demographic expansion, research addressing health behaviours within ethnic groups, particularly surrounding alcohol use among Korean immigrant women, remains limited. Understanding the sociocultural factors influencing alcohol consumption in this population is critical given the intersections of gender, culture, and acculturation pressures unique to immigrant women. Moreover, trends in South Korea show increasing alcohol use among women [8], which may have implications for those residing abroad. Investigating these patterns from a gender-specific and cultural perspective offers valuable insights for public health initiatives and culturally sensitive interventions.

This scoping review seeks to explore existing evidence and identify research gaps in current literature with respect to Korean immigrant women and alcohol use. A scoping review is effective for exploring broad, complex, or relatively under-researched areas where relevant empirical studies are scarce or scattered across various fields [9]. The purpose of this review is to map existing literature, identify knowledge gaps, clarify definitions, and describe how research has been conducted on a particular topic [9]. Considering the lack of studies on alcohol use in Korean immigrant women and the diversity in international research findings, a scoping review offers a comprehensive overview of both what is established and what remains unknown [9], making it a suitable methodology to meet the objectives of this review.

A general exploration of existing research on Korean individuals and alcohol use reveals an overwhelming focus on men. Several existing studies on Korean adult women and alcohol use have been conducted within South Korea [10–20] and the U.S. [21–28], that reveal a shifting landscape marked by increasing prevalence and distinct gender-specific trends. While historically lower than men, rates of alcohol consumption and harmful drinking among Korean women have risen significantly in recent years, reflecting broader cultural changes [10,18,19]. Women’s drinking behaviours are strongly influenced by social and psychological factors, frequently serving as coping strategies for stress, depression, and workplace gender discrimination [11,14,16]. Marital status and occupational settings further influence risk, with higher rates of harmful drinking observed among married women and those facing workplace inequality [15,16]. Gender-appropriate support systems and tailored interventions have been identified as vital in addressing relapse and dependence [11,12,17]. Among Korean immigrant women in the U.S., acculturation, religious beliefs, and social networks were found to influence both drinking behaviours and treatment engagement [21,23,26,29].

Current literature on alcohol use among Koreans in Canada focuses on either youth [29,30] or older adults (≥65 years) [31–33], while research addressing adults aged 25–65 years remains limited.

Although alcohol and substance use have been explored within various racialized communities in Western societies, there is a noticeable gap in primary research concerning the mental health and substance use experiences of Korean immigrant women. The increasing rate of alcohol use among Korean women (but not men) in South Korea is a concerning trend [8]. Similarly, in Canada, reported past-year alcohol use has remained constant among males since 2013 (approximately 80%) but has increased significantly among females [34]. Given these social changes and the substantial growth of the Korean immigrant population over the past 30 years [1], it is important to examine the factors related to alcohol use through cultural and gender-specific lenses.

### Objective

This scoping review sets out to explore the current state of evidence on Korean immigrant women and alcohol use and to identify potential gaps in knowledge.

## Materials and methods

### Protocol and registration

The five-stage framework described by Arksey and O’Malley and further refined by Levac et al. is applied in this scoping review [35]. Step 1 involved identifying the research question: “What is the current state of evidence on alcohol use among Korean immigrant women?” In Step 2, relevant studies were identified through a comprehensive, multi-database search strategy. In Step 3, articles were refined according to inclusion and exclusion criteria for title/abstract and full-text screening. In accordance with recommendations from Levac et al., a secondary independent reviewer confirmed the relevant articles to be included [36]. In Step 4, data were extracted and charted from eligible studies. In Step 5, findings were summarised and implications and limitations discussed. In Step 6, a plan for consulting with stakeholders and disseminating knowledge is included in order to link study findings with affected populations [36].

**Fig 1:**
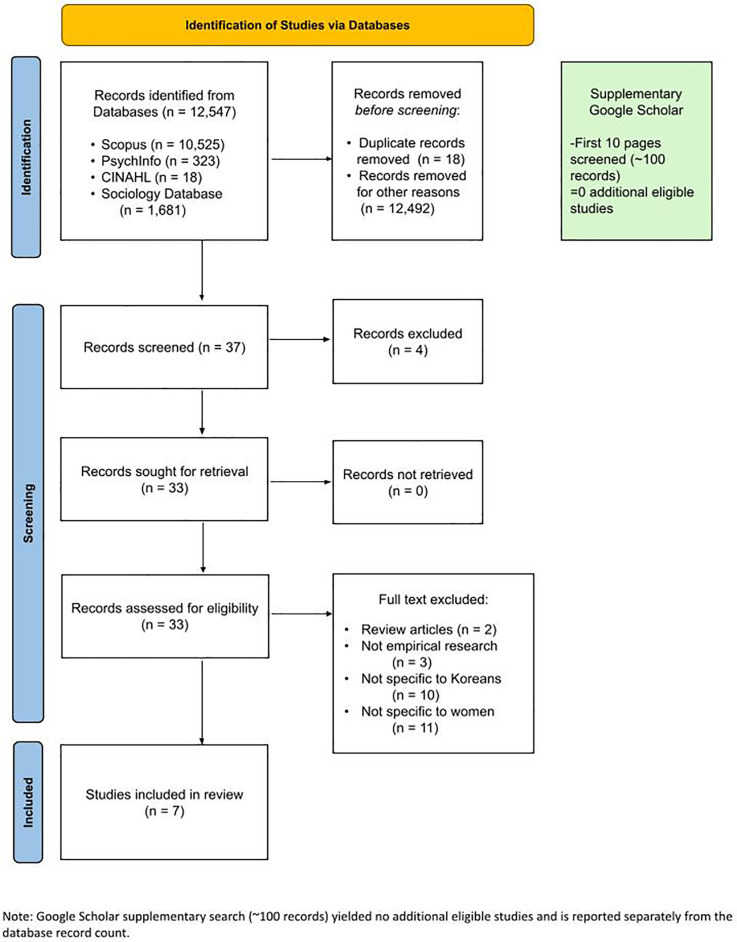
PRISMA Flowchart.

This scoping review applies the multi-step framework of the JBI-PRISMA protocol for reporting scoping reviews (Fig 1), using the PRISMA Extension for Scoping Reviews Checklist (Table 2) [37].

This study was prospectively registered on the Open Science Framework website at https://osf.io/fbx4n/.

### Eligibility criteria and rationale

- **Years considered:** All time. Due to the apparent gap in knowledge regarding this topic, date restrictions were not implemented. Eligible studies were expanded to include articles from all time periods in order to consider both earlier and more recent relevant research.
- **Language:** English only. Studies directed toward English-speaking audiences were included, as these best represent research oriented toward multicultural and international audiences.
- **Publication status:** Peer-reviewed primary articles published in academic journals were eligible for inclusion due to their commitment to rigour and validity.
- **Countries:** Studies from Canada, the U.S., the United Kingdom, Australia, and New Zealand were considered, reflecting English-speaking societies with established Korean immigrant communities.
- **Topic –** Studies in which alcohol use or alcohol-related behaviours were a central focus and in which Korean women formed a primary population of interest were included. Studies primarily focused on other topics (e.g., intimate partner violence, acculturation, stress, obesity) were excluded unless alcohol use among Korean women was clearly examined as a main outcome. Studies focusing on substances other than alcohol were excluded.
- **Population:** Studies were eligible if their samples comprised adult Korean women (≥18 years), or if the study context (e.g., community household or adult health surveys) indicated an adult population. Studies that explicitly focused on youth, students, or older adults (≥65 years) were excluded. In multi-ethnic studies or those including both men and women, articles were retained if Korean women constituted a reported analytic subgroup or if disaggregated findings for Korean women could be extracted.

One qualitative case study that included one male and one female Korean immigrant was included because the analytic focus and reported illustrations concerned gendered experiences of drinking; the limited fit with the population definition has been acknowledged as a limitation in the Discussion.

Women of Korean ethnicity residing in Canada, the United States, the United Kingdom, Australia, or New Zealand were the focus population. Eligible studies included Korean women who were first-generation immigrants (born in Korea and migrated to a host country) and studies where Korean ethnicity was reported but nativity or immigration generation were not specified. When studies combined foreign-born and host-country-born Korean women without disaggregated estimates, the study was retained if Korean women formed a clearly defined analytic subgroup and alcohol outcomes were reported for that subgroup. These limitations of the available population data are explicitly discussed in the Results and Discussion.

### Information sources

Four bibliographic databases were reviewed: Scopus, CINAHL, APA PsycInfo, and Sociology Database. Google Scholar was also used as a supplementary source to capture potentially relevant articles not indexed in these databases (further details are provided in the Search Details section). The most recent search of all sources took place on May 20, 2025.

### Search details

Keywords related to population (Korean, Korean immigrant, Korean women), alcohol-related behaviour (alcohol use, drinking, alcohol consumption), and geography (Canada, United States, United Kingdom, Australia, New Zealand) were combined into search strings using the Boolean operators “OR” and “AND.” For each database, filters were applied to restrict results to peer-reviewed journal articles, human participants (where available), and the English language. Where possible, field restrictions (e.g., title, abstract, keyword fields) were used to increase specificity, and strategies were iteratively refined based on preliminary results. No date limits were applied.

Although this review initially focused on the Canadian context, the very limited number of Canadian studies necessitated inclusion of research from other English-speaking countries with established Korean diasporic communities. These countries were selected because they share broadly comparable patterns of immigration, settlement, and cultural and linguistic diversity.

In Scopus, title, abstract, and keyword fields were searched using combinations of Korean identity, alcohol use, and country terms. A typical Scopus strategy was: (Korea* OR “Korean immigrant*” OR “Korean American*”) AND (“alcohol use” OR “alcohol consumption” OR drinking OR alcohol) AND (Canada OR “United States” OR “United Kingdom” OR Australia OR “New Zealand”), with filters applied for document type (peer-reviewed article) and language (English).

The search steps for Scopus are outlined below as an example:

- Basic search: Korea* AND women AND alcohol
- Results: 1,581 documents
- Limit results to: articles (type), English (language), Canada (country – subsequently modified to each of the other countries under consideration)
- Results: 12 articles
- A content scan of these 12 articles confirmed 0 relevant documents

Parallel strategies were used in CINAHL, APA PsycInfo, and Sociology Database. Where appropriate subject headings were available, they were combined with equivalent free-text terms using Boolean operators. Searches were conducted in title, abstract, keyword, and subject-heading fields, with limits applied to peer-reviewed journal articles, human participants (where available), and English language. Full database-specific strategies are presented in S1-S2 Appendix in S1 File.

Google Scholar was used as a supplementary source by screening the first 10 pages of results for core population and alcohol-related keywords. Potentially relevant records were exported and manually deduplicated against database results. Given the documented limitations of Google Scholar for evidence synthesis, including noisy and inconsistent indexing, constrained search options, and the prevalence of duplicates, it was not used as a primary source for study identification [38–42]. This approach aligns with methodological evaluations that have questioned the suitability of Google Scholar as a stand-alone source for systematic or scoping reviews while recognising its utility for identifying additional records [38,39].

### Selection of sources of evidence

Articles were considered for inclusion if they focused on alcohol use and Korean women as main subjects. After exporting all search results from the four databases, records were combined in a single Microsoft Excel spreadsheet, and exact duplicates were identified and removed through manual visual inspection of titles, authors, year, and journal. Google Scholar results (approximately 100 records from the first 10 pages) were similarly reviewed and deduplicated against database results. The resulting unique set of records formed the basis for title and abstract screening.

Articles must have stated focus on Korean women of adult age, living in one of the five eligible countries, in addition to the subject of alcohol use. Articles focused on substance use other than alcohol were excluded. Other excluded articles focused on Korean women and topics other than alcohol use, such as intimate partner violence, acculturation, stress, and obesity. In studies that included broader Asian subgroups, only findings relevant to Korean women were considered for inclusion.

Only peer-reviewed journal articles of primary studies were included. Non-peer-reviewed sources such as dissertations and community reports were excluded to ensure that knowledge generated through this review is based on consistent standards of quality, rigour, and credibility [9]. Title and abstract screening was performed independently by two reviewers (the first and second authors), with disagreements resolved through discussion to consensus. Full-text screening was conducted by the first author and confirmed by a secondary reviewer, with disagreements resolved through discussion.

Key findings from articles that addressed the main focuses of Korean women and alcohol use were identified and extracted. Qualitative thematic analysis was conducted using an inductive approach as outlined by Gottlieb et al. (2021) [43]. The list of themes was iteratively refined and expanded throughout the data extraction process, with previous analyses updated accordingly. Resulting themes and findings were subsequently reviewed by three additional researchers with relevant domain expertise.

### Critical appraisal of individual sources of evidence

Consistent with our scoping review objective to map and describe the existing evidence rather than formally evaluate intervention effectiveness, a structured critical appraisal of individual studies was not conducted. This approach aligns with foundational scoping review guidance, which positions quality assessment as optional and dependent on review purpose [35]. Nonetheless, we comment qualitatively on methodological features (e.g., sampling frames, outcome measures) where they bear directly on confidence in particular findings.

## Results

### Selection of sources of evidence

The four bibliographic databases yielded 12,547 records in total. A supplementary Google Scholar search screened the first 10 pages of results (approximately 100 records); no additional eligible studies were identified beyond those obtained from the database searches. After duplicate removal and removal for other reasons, 37 records remained for title and abstract screening. Of these, 33 were assessed for full-text eligibility, and seven articles met the inclusion criteria. Reasons for exclusion at the full-text stage included lack of focus on Korean women, alcohol use not being a main subject, ineligible age group, or non-primary/non-peer-reviewed publication type.

A summary of inductively-determined themes and findings from each article was tabulated (Table 1). Three major themes associated with influences on alcohol use among Korean American women were identified: acculturation, religion, and social networks. As noted in the eligibility criteria, some included studies comprise Korean-American women with unspecified nativity or combined foreign-born and U.S.-born samples; findings should therefore be interpreted as applying to Korean women in the U.S. more broadly, with implications for Korean immigrant women specifically.

**Table 1:** Summary of findings.

Reference #	Citation and Country	Objective	Study Design	Population	Sex Composition	Alcohol Outcomes	Rationale for Inclusion	Themes
21	Ayers, et al., 2011. USA	To estimate the association between cultural and social mechanisms and Korean-American women’s drinking behaviours	Cross‑sectional household survey	591 Korean women aged ≥18 years, identified via random sampling of households with Korean surnames; sample included both foreign‑born and U.S.‑born Korean Americans	Female only	Any drinking in past year; frequency of drinking; heavy episodic drinking.	Large, community‑based sample of Korean women with disaggregated alcohol outcomes, directly focused on drinking behaviours	Social networks (drinking models, drinking support), acculturation indicators (language use, media preferences)
22	Ayers et al., 2009, USA	To identify social reinforcers within religious institutions associated with alcohol consumption among Korean women in California	Cross‑sectional household survey	591 Korean women aged ≥18 years in California, drawn from the same sampling frame as Ayers et al., 2011; included both foreign‑born and U.S.‑born Korean Americans (nativity not disaggregated)	Female only	Any alcohol use; frequency of use; heavy drinking	Direct focus on alcohol use among Korean women and religious contexts within the same community‑based sample	Religion (denomination, religious attendance, congregational messages discouraging drinking); social influence of fellow congregants vs religious leaders
23	Lee, 2022. USA	To identify types of coping strategies used by Korean immigrant women in USA	Cross-sectional study	136 first‑generation Korean immigrant women residing in Texas (born in Korea; immigrated as adults)	Female only	Frequency of alcohol use; use of alcohol as a coping strategy	Only recent study focused specifically on Korean immigrant women’s coping and alcohol; provides detailed acculturation‑related measures	Psychological and behavioral acculturation, coping, alcohol use
25	Maxwell, et al., 2012. USA	To assess the prevalence of excess body weight, physical inactivity and alcohol and tobacco use in 5 Asian-American subgroups	Cross‑sectional population‑based telephone survey (California Health Interview Survey)	3,465 Asian American adults in California (1,285 Chinese, 421 Japanese, 620 Korean, 659 Filipino, 480 Vietnamese); mixed‑sex sample; Korean women constitute one analytic subgroup (estimates not stratified by immigration generation)	Men and women; Korean female subgroup analysed separately	Any alcohol use; binge drinking (≥5 drinks on one occasion in past month); current smoking; other health risk behaviours	Provides disaggregated alcohol outcomes for Korean women within a large representative survey, enabling comparison with other Asian subgroups	Health risk behaviour prevalence across Asian American subgroups; comparative binge‑drinking rates for Korean American women relative to other Asian American groups
26	Ryu, et al., 2013. USA	To compare health behaviours of Koreans in Korea and Koreans in America	Cross‑sectional analysis of two population‑based surveys (KCHS and CHIS)	Adults ≥19 years from the 2009 Korean Community Health Study (KCHS; n ≈ 230,715) and Korean‑American adults ≥18 years from the 2009 California Health Interview Study (CHIS; n = 923); analyses include Korean American women as a subgroup, with foreign‑born and U.S.‑born respondents combined	Men and women; Korean American female subgroup analysed	Any alcohol use; frequency and quantity of alcohol consumption; binge drinking	Allows comparison of alcohol patterns between Korean women in Korea and Korean‑American women, with acculturation indicators	Acculturation (length of U.S. residence, language used at home, self‑identified cultural orientation); cross‑national differences in drinking patterns between Koreans in Korea and Korean Americans
27	Sim, et al., 2013. USA	To assess the association between religious denomination and alcohol intake among Korean American women	Cross‑sectional interview study	Korean American women in California (sample stratified across three Christian denominations); adult women ≥18 years; nativity (foreign‑ vs U.S.‑born) not consistently disaggregated	Female only	Any drinking; heavy drinking; drinking frequency	Focus on alcohol intake among Korean American women in relation to religious denomination and practice	Religion (denominational affiliation, religious attendance); religious norms regarding alcohol; interplay between religiosity and drinking behaviours
28	Yun & Park, 2008. USA	To explore the characteristics of alcohol consumption by first generation Koreans who immigrated to the USA after age 18	Qualitative case study	2 Korean adult immigrants (1 man, 1 woman) who migrated to the U.S. after age 18	Mixed: one male and one female participant	Narrative accounts of drinking context, occasions, and perceived consequence	Included because it provides rich qualitative detail on gendered drinking experiences of the female participant and sociocultural drinking contexts, despite small sample and mixed‑sex design	Social networks, cultural expectations at celebrations, proximity to Korean drinking venues

### Acculturation

Three of the seven articles focused on acculturation, employing differing methodologies and producing conflicting results. Ayers et al. (2011) evaluated acculturation by examining language spoken at home, English proficiency, media preferences, and the ethnic composition of social networks [21]. Women were grouped as traditional, bicultural, or acculturated based on their levels of engagement with American versus Korean cultural norms. This study found no significant relationship between alcohol use and acculturation levels among Korean-American women [21].

In contrast, Lee (2022) approached acculturation through a psychological and behavioural lens, considering cultural identity, adaptation-related stress, and emotional integration in addition to observable behaviours such as language use and social networks [23]. This study found that Korean-American women with higher levels of acculturation consumed more alcohol compared to those who were less acculturated [23].

Ryu et al. (2013) employed a comparative framework involving Korean Americans in California and Koreans living in South Korea [26]. Variables including language used at home, length of U.S. residence, and self-identified cultural orientation were measured. Findings revealed that 57% of traditional, 54% of bicultural, and 79% of acculturated Korean-American women reported alcohol use; the proportion of binge drinkers was twice as high in the acculturated group compared with the traditional and bicultural groups [26].

These inconsistent findings indicate the complexity of the concept of acculturation and underscore the need for further research to better understand its impact on alcohol consumption.

### Religion

Two of the seven articles discussed the influence of religion on alcohol consumption among Korean women living in California. Sim et al. (2013) found that women with no religious affiliation showed the highest probability of being heavy drinkers [27]. Among Christian denominations, being Catholic was associated with a fivefold greater probability of alcohol consumption, while women in Conservative Christian groups indicated the lowest levels of consumption [27].

Ayers et al. (2009) compared the influence of alcohol-discouraging messages delivered by either religious leaders or fellow church members, finding that the social influence of other congregants was significantly associated with the probability of alcohol consumption, whereas the influence of religious leaders did not show a significant effect [22]. It remains unclear whether similar messages from individuals outside the church would have a comparable or lesser effect; a question that relates to the social network influences discussed in the following section.

These findings suggest that religious affiliation and congregational social influences may be associated with alcohol use among Korean American women in these samples, though the evidence base is limited to two California-based studies and warrants cautious interpretation.

### Social networks

Several articles revealed associations between social networks and alcohol consumption in Korean women. Ayers et al. (2011) found that social networks had more influence on alcohol use behaviours than levels of acculturation [21]. Specifically, social connections, particularly relationships with siblings and friends who share similar drinking behaviours, had a stronger influence on alcohol consumption than acculturation alone [21].

A case study by Yun and Park (2008) offers illustrative rather than generalizable evidence, given its two-person sample including one male participant; findings should be interpreted with corresponding caution. With that caveat, the study describes how cultural expectations influenced alcohol use; social settings such as a baby’s 100th-day birthday celebration (known as *baek-il*) encouraged alcohol consumption, with guests reporting feeling compelled to drink more to demonstrate happiness and support [28]. Proximity to Korean bars and alcohol retail outlets also strongly influenced alcohol use [28].

Moreover, binge drinking prevalence in the last 30 days among Korean American women (12%) was roughly 4 times higher than among Chinese American women (3%) [25], indicating markedly higher relative prevalence despite low absolute rates in all groups. This pattern highlights the importance of analysing Asian Americans in their distinct subgroups so that within-group differences in alcohol-related risk are not obscured.

## Discussion

Despite the paucity of research on alcohol use among Korean women in Western societies, evidence from the U.S. provides insight into key patterns within this population. Three primary sociocultural domains – acculturation, religion, and social networks – have emerged as influences on drinking behaviours among Korean immigrant women.

The evidence on acculturation is inconclusive. While some studies found no significant association between acculturation level and alcohol use [21], others reported that higher levels of acculturation are correlated with increased alcohol and tobacco use [23]. Contributing factors include younger age, peer endorsement of drinking, exposure to social models who consume alcohol, and direct encouragement from friends [24]. Korean-American women show higher rates of binge drinking compared with other Asian American groups [25], indicating diversity within the broader Asian-American population.

The seven included studies did not explicitly conceptualize or measure settler colonialism, racial hierarchies, or White cultural dominance. Instead, these constructs are drawn from broader critical scholarship to contextualise the environments in which Korean immigrant women live and drink [44–46]. From this perspective, acculturation processes unfold within White settler societies where racialized immigrants navigate unequal power relations, systemic racism, and expectations of conformity to dominant norms, rather than in a neutral space of cultural exchange [44,45]. Immigrant women experience additional pressures shaped by intersecting racial and gendered colonial norms [46]. Thus, acculturation within White settler societies is a complex, power-driven process embedded in ongoing colonial legacies and racial inequalities. We therefore interpret the observed associations between acculturation, social networks, and alcohol use in dialogue with this theoretical literature, while recognising that the primary studies themselves did not directly investigate these structural dimensions.

The inconsistent findings on acculturation indicate that further research is necessary to better understand its impact on alcohol consumption among Korean immigrant women living abroad. Since each of the three articles on acculturation employed distinct measurement variables, exploring context-specific methods for understanding and evaluating acculturation will be valuable for promoting greater consistency and depth in future research.

Religion represents another significant factor influencing alcohol consumption in Korean women. Throughout the 20th century, religion became a momentous part of Korean women’s lives as South Korea experienced rapid Christianization, fuelled by missionary efforts, modernisation, and the active involvement of churches in education and social welfare [47]. Christianity provided Korean women with new social opportunities, leadership roles, and emotional support, enabling them to form strong community networks both within Korea and abroad. As central social institutions, churches influenced daily life by promoting community cohesion and offering spaces for emotional and practical assistance, deeply shaping social ties and identity, particularly for Korean women living overseas [48].

The finding that women without religious affiliation consistently show higher alcohol use, and that Catholic Korean-American women in California have the highest likelihood of drinking among Christian denominations [27], highlights the need for further research to understand the factors driving these differences across religious groups. Moreover, the influence of fellow congregants was closely linked to the likelihood of alcohol consumption while the influence of religious leaders did not appear to affect drinking behaviour [22], further underscoring the role of social networks.

Our interpretation that ethnic and religious networks may serve both as sources of social support and as contexts for alcohol use draws on theoretical and empirical work beyond the seven included studies. While the primary evidence documents associations between congregational influences, peer networks, and drinking patterns, it does not systematically examine how these networks are shaped by racialization, gendered expectations, or colonial histories. These structural considerations are therefore presented as a complementary lens rather than as empirically demonstrated mechanisms.

Cultural expectations influenced alcohol consumption at Korean celebrations, where drinking is integrated into social rituals that emphasise respect, solidarity, and communal cohesion [28]. Alcohol is frequently present at significant cultural events such as ancestor memorial ceremonies, weddings, and seasonal festivals, representing symbolic practices that honour cultural heritage while reinforcing collective identity [28]. These occasions are governed by formal drinking etiquettes rooted in Confucian principles stressing social hierarchy, filial piety, and group harmony [49,50]. The cultural concept of *jeong* (emotional-affective attachment) fosters practices of generosity and reciprocity in drinking interactions, reinforcing interpersonal bonds but also contributing to patterns of overconsumption in some contexts [49]. As such, alcohol use in celebratory contexts transcends mere intoxication, serving as a culturally meaningful mechanism for sustaining social norms and emotional connectivity.

Additionally, proximity to Korean bars and alcohol retail outlets, as well as proximity to others who consume alcohol, has been cited as influencing Korean American women’s alcohol use [28]. Societal differences between South Korea and the U.S. were also identified, particularly regarding the significantly stricter DUI laws in the U.S., which appeared to catch Korean individuals off guard when apprehended [28].

Only a subset of included studies referred to immigration-related stressors (for example, difficulties adjusting to the host environment, economic pressures, and language barriers) in relation to alcohol use [23,28]. Because these stressors were not consistently operationalised or examined as primary outcomes, any conclusions regarding immigration stress remain tentative. Immigration-related stress is therefore interpreted as a plausible contributing context, rather than a well-established determinant, highlighting the need for future research that explicitly measures and theorises this construct among Korean immigrant women.

These findings highlight the social, cultural, and relational factorsthat have been documented in relation to alcohol use within this population. It is important to distinguish three categories of knowledge here: (1) findings directly supported by the seven included studies (the roles of acculturation, religion, and social networks); (2) plausible contextual hypotheses generated from broader theoretical and empirical literature (e.g., the potential influence of racialization, patriarchal norms, and immigration stress); and (3) priorities for future primary research. It remains crucial to investigate whether similar patterns exist among Korean women in Canada, especially as heavy alcohol consumption continues to rise among women in both Canada and South Korea. Future studies should also directly examine intersectional factors including racial discrimination, family relationships, and patriarchal norms that remain largely unexplored in the available primary research.

### Implications

Several important implications arise from this scoping review. There is a clear need to address the knowledge gap concerning alcohol use among Korean immigrant women. Existing research has been conducted exclusively in the U.S., despite this study’s exploration of other culturally similar and demographically diverse countries.

Engagement in heavy alcohol use continues to rise among women while that of men remains relatively stable, both in Canada [34] and in South Korea [8]. This gender convergence trend is concerning for public health, as women generally experience different and greater health risks from heavy alcohol use compared with men [51]. In addition, Sim et al. (2013) found that Korean-American women have the highest rate of alcohol consumption among all Asian American Pacific Islander groups, a finding that challenges the ‘model minority’ stereotype and underscores that certain subgroups may be more vulnerable than others [27,52,53]. In the absence of research and contextual knowledge, the development of effective public health promotion and recovery programmes remains severely constrained.

It would be prudent to conduct further investigations into the intersectional influences on alcohol use in Korean immigrant women, as existing studies have ignored topics such as discrimination, spousal or family conflict, and patriarchal oppression. Other literature focused on Asian Americans more broadly has cited racial discrimination as having strong influences on alcohol use [52–56]. Ethnic minorities have identified racial discrimination as highly stressful life experiences, and among Asian Americans, racial discrimination and self-reported negative treatment from others has been correlated with elevated levels of alcohol consumption [53]. Fear of contributing to negative stereotypes about one’s own racialized community has also been found to contribute to the likelihood of engaging in alcohol use [53].

Within the family sphere, traditional Korean norms emphasising the preservation of family honour and the concealment of difficulties may intensify conflict and discourage open communication [57]. A culturally embedded societal expectation prescribes that individual suffering and potentially negative portrayals of oneself are to remain hidden or resolved internally [57], which may lead women to use alcohol as a means of escape [58]. Indeed, research has found that individuals who internalise stressors report significantly greater alcohol-related difficulties than control groups [59].

Korean culture, similar to other East Asian cultures, is permeated with norms of respect, patriarchy, and filial piety, all of which continue to influence South Korean society despite rapid modernisation [60,61]. Korean patrilineal norms typically involve married couples co-residing with the husband’s parents, with the oldest son and his wife bearing the greatest obligation [62]. Such societal systems continue to undervalue the perspectives and experiences of women and contribute to violence against women [63]. Scholars have argued that cultural norms such as filial piety and patriarchy reinforce the oppression of women and maintain power differentials and gender inequalities [64,65], which in turn may generate stressors plausibly linked to alcohol use and may create significant barriers to accessing support or treatment [58]. None of the seven included studies directly measured these dynamics, so these connections are offered as contextual hypotheses warranting empirical investigation, rather than as established findings of this review.

With regard to service utilisation, Korean immigrant women are likely to avoid seeking treatment or services, particularly if they ascribe to cultural notions that portraying mental health concerns may be shameful to the family [27,54,66]. Language barriers and other structural factors such as inability to take time off work and childcare responsibilities further compound this avoidance [54]. Korean women who become dependent on alcohol often feel tremendous amounts of shame related to their perceived failures and their inability to fulfill expected gender roles [14], which in turn are likely to contribute to relapse [64,65].

Collectively, these intersecting cultural and social forces reflect broader patterns of immigrant health inequities, shaped by structural racism, gendered power imbalances, and systemic barriers within healthcare. Addressing alcohol use among Korean immigrant women therefore requires prevention and intervention strategies that are not only gender-responsive and culturally sensitive but also attentive to the broader structural determinants of immigrant health.

### Knowledge dissemination

This scoping review is aligned with a larger research project on Korean Canadian women with alcohol use concerns who reside in Toronto, Canada, being conducted by the same principal investigator. The research involves a phase whereby participants are invited to a group engagement meeting, where knowledge sharing and dissemination will take place. This step, which was not part of Arksey and O’Malley’s original five-stage framework [35], was introduced and strongly endorsed by Levac et al. [36] as an essential addition that supports meaningful knowledge translation.

In alignment with Levac et al.’s recommendation [36], the findings from this scoping review will be shared with participants of the research project, who represent those most directly affected by this research. This consultation phase will enable participants to provide feedback, share insights, and help interpret the findings in a way that reflects their lived experience. The group engagement meeting serves not only as a dissemination activity but also as an opportunity for co-creation of knowledge, where the similarities and differences between themes from this scoping review and the ongoing research project will be discussed.

Stakeholder engagement is critical for enriching the review’s relevance and applicability. The feedback and insights gathered from participants will be integrated into the manuscript of the final research project, thereby ensuring that their perspectives inform the interpretation of findings and the direction of future research.

### Limitations

This scoping review has several limitations that should be acknowledged. The limited number of articles (seven) and the relatively dated nature of the articles (six published before 2014, while one was published in 2022), suggest a dearth of up-to-date knowledge on Korean women and alcohol use. All of the articles included in this review were published in the U.S., suggesting that other countries, including Canada, have yet to investigate this important mental health topic.

As all of the articles examined were published in the U.S., it was not possible to identify any sociocultural influences on alcohol use that are specific to the Canadian context. For example, the younger legal drinking age in Canada (19 versus 21 years in the U.S.) and differences in healthcare systems, are variations from neighbouring countries that may be impactful.

The included studies rarely distinguished first‑ from later‑generation Korean women, which constrained our ability to compare patterns by immigration generation.

This scoping review does not attempt to assess the quality or rigour of existing evidence, but rather to map the overall state of research knowledge, which is one of the key reasons for conducting scoping studies, as noted by Arksey & O’Malley [35]. In contrast to systematic reviews, which typically address narrowly defined questions often regarding the effectiveness of interventions or quantitative data synthesis, scoping reviews accommodate a wide variety of evidence types, research methods, and study designs [9].

A further limitation is that a standardized critical appraisal of included studies was not engaged in throughout this scoping review. Given the very small and heterogeneous evidence base, the assumption was that applying multiple appraisal tools would yield limited additional insight beyond our narrative assessment. However, this choice restricts the ability to comment on the relative robustness of individual studies, and future reviews could usefully incorporate formal quality assessment as more primary research accumulates.

Finally, this review did not incorporate formal citation chaining or systematic screening of reference lists in addition to database and supplementary Google Scholar searches. Although potentially relevant articles encountered incidentally during full-text review were noted, these were not formally screened or included as part of the search process. As a result, it is possible that some relevant studies were not identified. Future reviews in this area should include structured citation chasing as a complementary strategy to enhance retrieval completeness.

## Conclusion

This scoping review sought to identify and examine the sociocultural factors influencing alcohol use among Korean immigrant adult women. The findings reveal a significant gap in knowledge, with no research conducted within Canada or addressing the Canadian context. A search for studies from countries with diverse yet comparable contexts identified seven relevant articles from the United States.

As global populations continue to diversify, it becomes increasingly important to address mental health and substance use challenges with approaches that are both gender-sensitive and culturally appropriate. Considering the rising alcohol consumption rates among women relative to men in recent decades, along with notably higher alcohol use reported among Korean communities compared with other Asian groups in the U.S., understanding the unique experiences shaped by culture and gender is essential to developing effective, relevant interventions and support for Korean immigrant women.

## Supporting information

S1 File Appendices. (DOCX)
