# Supplementary material for: Sociocultural factors influencing alcohol use among Korean immigrant women: A scoping review
Source: PLoS One. 2026 Jul 14;21(7):e0353742. doi: 10.1371/journal.pone.0353742 (PMC13367703; doi:10.1371/journal.pone.0353742)
Supplement: S1 File — (DOCX) [file pone.0353742.s001.docx]

# **S1 Appendix. Database-specific Search Strategies**

**S1 Appendix. Database-Specific Search Strategies**

The following tables present the complete executed search strategies for each database and supplementary source, including all keyword strings as run and the number of records retrieved per string. Limits applied across all databases: English language, peer-reviewed journal articles, all dates. Search strategies were iteratively refined based on preliminary results; where available, field restrictions (title, abstract, keyword) were applied to increase specificity. The search date for each database is noted below.

**Scopus**

Date searched: May 20, 2025

Limits: English language, peer-reviewed articles, all dates

Fields searched: Title, Abstract, Keywords (TITLE-ABS-KEY)

| **Search string (executed)** | **Records retrieved** |
| --- | --- |
| ***Canada*** | |
| Korea* women Canad* alcohol | 2,649 (all excluded at title/abstract screening) |
| Korea* Canad* alcohol | 5,875 (all excluded at title/abstract screening) |
| Korean alcohol Canada | 12 (all excluded at title/abstract screening) |
| Asia* alcohol Canada | 1,829 (all excluded at title/abstract screening) |
| ***United States*** | |
| Korea* women (USA OR America*) alcohol | 17 (16 after deduplication; 1 excluded; 0 eligible) |
| Korea* (USA OR America*) alcohol | 71 (0 new after deduplication) |
| Asia* alcohol America | 7 (0 new after deduplication) |
| ***United Kingdom*** | |
| Korea* women (England OR Britain OR UK) alcohol | 8 (0 new after deduplication) |
| Korea* (England OR Britain OR UK) alcohol | 37 (0 new after deduplication) |
| Korean alcohol (England OR Britain OR UK) | 6 (0 new after deduplication) |
| ***Australia / New Zealand*** | |
| Korea* women (Australia OR "New Zealand") alcohol | 7 (0 new after deduplication) |
| Korea* (Australia OR "New Zealand") alcohol | 4 (0 new after deduplication) |
| Korean alcohol (Australia OR "New Zealand") | 3 (0 new after deduplication) |

*Note: The search description provided in the manuscript body ("Basic search: Korea* AND women AND alcohol → 1,581 results, limited to 12 articles") was an illustrative example of the limit-application step and has been replaced in this revised appendix with the full executed strategy documented above.*

**CINAHL**

Date searched: March 10, 2025

Limits: English language, peer-reviewed articles, all dates, human participants

Fields searched: Title, Abstract, Subject Headings

| **Search string (executed)** | **Records retrieved** |
| --- | --- |
| ***Canada*** | |
| Korea* women Canad* alcohol | 0 |
| Korea* Canad* alcohol | 0 |
| Korean alcohol Canada | 0 |
| Asia* alcohol Canada | 0 |
| ***United States*** | |
| Korea* women (USA OR America*) alcohol | 2 (1 after deduplication; 1 excluded — comparison study not focused on alcohol as primary outcome among Korean women; 0 eligible) |
| Korea* (USA OR America*) alcohol | 15 (0 new after deduplication) |
| Asia* alcohol America | 1 (0 new after deduplication) |
| ***United Kingdom*** | |
| Korea* women (England OR Britain OR UK) alcohol | 0 |
| Korea* (England OR Britain OR UK) alcohol | 0 |
| Korean alcohol (England OR Britain OR UK) | 0 |
| ***Australia / New Zealand*** | |
| Korea* women (Australia OR "New Zealand") alcohol | 0 |
| Korea* (Australia OR "New Zealand") alcohol | 0 |
| Korean alcohol (Australia OR "New Zealand") | 0 |

**APA PsycInfo**

Date searched: May 20, 2025

Limits: English language, peer-reviewed articles, all dates, human participants

Fields searched: Title, Abstract, Keywords

| **Search string (executed)** | **Records retrieved** |
| --- | --- |
| ***Canada*** | |
| Korea* women Canad* alcohol | 23 (all excluded) |
| Korea* Canad* alcohol | 84 (83 after deduplication; all excluded) |
| Korean alcohol Canada | 18 (all excluded) |
| Asia* alcohol Canada | 122 (all excluded) |
| ***United States*** | |
| Korea* women (USA OR America*) alcohol | 0 |
| Korea* (USA OR America*) alcohol | 0 |
| Asia* alcohol America | 76 (0 eligible) |
| ***United Kingdom*** | |
| Korea* women (England OR Britain OR UK) alcohol | 0 |
| Korea* (England OR Britain OR UK) alcohol | 0 |
| Korean alcohol (England OR Britain OR UK) | 0 |
| Asia* alcohol (England OR Britain OR UK) | 0 |
| ***Australia / New Zealand*** | |
| Korea* women (Australia OR "New Zealand") alcohol | 0 |
| Korea* (Australia OR "New Zealand") alcohol | 0 |
| Korean alcohol (Australia OR "New Zealand") | 0 |
| Asia* alcohol (Australia OR "New Zealand") | 0 |

**Sociology Database**

Date searched: May 3, 2025

Limits: English language, peer-reviewed articles, all dates

Fields searched: Title, Abstract, Keywords

| **Search string (executed)** | **Records retrieved** |
| --- | --- |
| ***Canada*** | |
| Korea* women Canad* alcohol | 29 (0 eligible) |
| Asia* alcohol Canada | 133 (0 eligible) |
| ***United States*** | |
| Korea* women (USA OR America*) alcohol | 301 (1 potentially relevant: Yoo et al., 2010 — excluded, alcohol not primary focus among Korean women) |
| Korea* (USA OR America*) alcohol | 0 |
| Asia* alcohol America | 1,007 (2 potentially relevant: Ryu et al. [included]; Lee et al. 2015 [excluded — not focused on alcohol as primary outcome for Korean women]) |
| ***United Kingdom*** | |
| Korea* women (England OR Britain OR UK) alcohol | 18 (0 eligible) |
| Korea* (England OR Britain OR UK) alcohol | 0 |
| Korean alcohol (England OR Britain OR UK) | 0 |
| Asia* alcohol (England OR Britain OR UK) | 98 (0 eligible) |
| ***Australia / New Zealand*** | |
| Korea* women (Australia OR "New Zealand") alcohol | 18 (0 eligible) |
| Korea* (Australia OR "New Zealand") alcohol | 0 |
| Korean alcohol (Australia OR "New Zealand") | 0 |
| Asia* alcohol (Australia OR "New Zealand") | 77 (0 eligible) |

**Google Scholar (Supplementary Search)**

Date searched: April 14, 2025

Limits: English language, peer-reviewed only (filter applied)

Method: First 10 pages of results (~100 records) manually reviewed per keyword set.

Google Scholar was used as a supplementary source only, given its documented limitations for systematic evidence synthesis including unstable hit counts, constrained search options, and high prevalence of duplicates. Raw Google Scholar hit counts are algorithmically generated estimates and were not processed as individual records; only the results visible on the first 10 pages per keyword set were actively reviewed. The table below reports the number of records actually reviewed, not the total hit count returned by the search engine. No additional eligible studies beyond those identified through the four primary databases were found through Google Scholar.

| **Search string (executed)** | **Records retrieved** |
| --- | --- |
| ***Canada-focused keyword sets*** | |
| Korea* women Canad* alcohol | First 10 pages (~100 results) reviewed; 6 records assessed for relevance; 0 eligible (dissertations, theses, and non-alcohol-focused articles excluded) |
| Korean Canadian women alcohol | First 10 pages reviewed; 3 full texts assessed; 0 eligible |
| Korean alcohol Canada | First 10 pages reviewed; 0 eligible |
| ***United States-focused keyword sets*** | |
| Korea* women (USA OR America*) alcohol | First 10 pages reviewed; 3 records assessed; 0 new eligible studies (all already identified through database searches) |
| Asia* alcohol America | First 10 pages reviewed; 0 new eligible studies |
| ***United Kingdom keyword sets*** | |
| Korea* alcohol (England OR Britain OR UK) | First 10 pages reviewed; 0 eligible |
| Asia* alcohol (England OR Britain OR UK) | First 10 pages reviewed; 0 eligible |
| ***Australia / New Zealand keyword sets*** | |
| Korea* alcohol (Australia OR "New Zealand") | First 10 pages reviewed; 0 eligible |
| Asia* alcohol (Australia OR "New Zealand") | First 10 pages reviewed; 0 eligible |

**S2 Appendix: Preferred Reporting Items for Systematic reviews and Meta-Analyses extension for Scoping Reviews (PRISMA-ScR) Checklist**

| **SECTION** | **ITEM** | **PRISMA-ScR CHECKLIST ITEM** | **ON PAGE #** |
| --- | --- | --- | --- |
| TITLE | | | |
| Title | 1 | Identify the report as a scoping review. | 1 |
| ABSTRACT | | | |
| Structured summary | 2 | Provide a structured summary that includes (as applicable): background, objectives, eligibility criteria, sources of evidence, charting methods, results, and  conclusions that relate to the review questions and objectives. | 3 |
| INTRODUCTION | | | |
| Rationale | 3 | Describe the rationale for the review in the context of what is already known. Explain why the review questions/objectives lend themselves to a scoping review approach. | 4 |
| Objectives | 4 | Provide an explicit statement of the questions and objectives being addressed with reference to their key elements (e.g., population or participants, concepts, and  context) or other relevant key elements used to conceptualize the review questions and/or objectives. | 5 |
| METHODS | | | |
| Protocol and registration | 5 | Indicate whether a review protocol exists; state if and where it can be accessed (e.g., a Web address); and if available, provide registration information, including the registration number. | 5 |
| Eligibility criteria | 6 | Specify characteristics of the sources of evidence used as eligibility criteria (e.g., years considered, language,  and publication status), and provide a rationale. | 5 |
| Information sources* | 7 | Describe all information sources in the search (e.g., databases with dates of coverage and contact with authors to identify additional sources), as well as the date the most recent search was executed. | 5 |
| Search | 8 | Present the full electronic search strategy for at least 1  database, including any limits used, such that it could be repeated. | 5 |
| Selection of sources of evidence† | 9 | State the process for selecting sources of evidence (i.e., screening and eligibility) included in the scoping review. | 6 |
| Data charting process‡ | 10 | Describe the methods of charting data from the included sources of evidence (e.g., calibrated forms or forms that have been tested by the team before their use, and whether data charting was done independently or in  duplicate) and any processes for obtaining and confirming data from investigators. | 7 |
| Data items | 11 | List and define all variables for which data were sought and any assumptions and simplifications made. | 7 |
| Critical appraisal of individual sources of evidence§ | 12 | If done, provide a rationale for conducting a critical appraisal of included sources of evidence; describe the  methods used and how this information was used in any data synthesis (if appropriate). | 7 |
| Synthesis of results | 13 | Describe the methods of handling and summarizing the data that were charted. | 7 |

| **SECTION** | **ITEM** | **PRISMA-ScR CHECKLIST ITEM** | **ON PAGE #** |
| --- | --- | --- | --- |
| RESULTS | | | |
| Selection of sources of evidence | 14 | Give numbers of sources of evidence screened, assessed for eligibility, and included in the review, with  reasons for exclusions at each stage, ideally using a flow diagram. | 8 |
| Characteristics of sources of evidence | 15 | For each source of evidence, present characteristics for which data were charted and provide the citations. | 9 |
| Critical appraisal within sources of evidence | 16 | If done, present data on critical appraisal of included sources of evidence (see item 12). | n/a |
| Results of  individual sources of evidence | 17 | For each included source of evidence, present the  relevant data that were charted that relate to the review questions and objectives. | 9 |
| Synthesis of results | 18 | Summarize and/or present the charting results as they relate to the review questions and objectives. | 9 |
| DISCUSSION | | | |
| Summary of evidence | 19 | Summarize the main results (including an overview of concepts, themes, and types of evidence available), link to the review questions and objectives, and consider the relevance to key groups. | 10 |
| Limitations | 20 | Discuss the limitations of the scoping review process. | 11 |
| Conclusions | 21 | Provide a general interpretation of the results with respect to the review questions and objectives, as well  as potential implications and/or next steps. | 11 |
| FUNDING | | | |
| Funding | 22 | Describe sources of funding for the included sources of evidence, as well as sources of funding for the scoping review. Describe the role of the funders of the scoping review. | 13 |

JBI = Joanna Briggs Institute; PRISMA-ScR = Preferred Reporting Items for Systematic reviews and Meta-Analyses extension for Scoping Reviews.

* Where *sources of evidence* (see second footnote) are compiled from, such as bibliographic databases, social media platforms, and Web sites.

† A more inclusive/heterogeneous term used to account for the different types of evidence or data sources (e.g., quantitative and/or qualitative research, expert opinion, and policy documents) that may be eligible in a scoping review as opposed to only studies. This is not to be confused with *information sources* (see first footnote).

‡ The frameworks by Arksey and O’Malley (6) and Levac and colleagues (7) and the JBI guidance (4, 5) refer to the process of data extraction in a scoping review as data charting*.*

§ The process of systematically examining research evidence to assess its validity, results, and relevance before using it to inform a decision. This term is used for items 12 and 19 instead of "risk of bias" (which is more applicable to systematic reviews of interventions) to include and acknowledge the various sources of evidence that may be used in a scoping review (e.g., quantitative and/or qualitative research, expert opinion, and policy document).

*From:* Tricco AC, Lillie E, Zarin W, O'Brien KK, Colquhoun H, Levac D, et al. PRISMA Extension for Scoping Reviews (PRISMA- ScR): Checklist and Explanation. Ann Intern Med. ;169:467–473. doi: 10.7326/M18-0850
